# Metabolic signature and proteasome activity controls synovial migration of *CDC42^hi^* CD14^+^ cells in rheumatoid arthritis

**DOI:** 10.3389/fimmu.2023.1187093

**Published:** 2023-08-17

**Authors:** Eric Malmhäll-Bah, Karin M.E. Andersson, Malin C. Erlandsson, Sofia T. Silfverswärd, Rille Pullerits, Maria I. Bokarewa

**Affiliations:** ^1^ Department of Rheumatology and Inflammation Research, Institute of Medicine, University of Gothenburg, Gothenburg, Sweden; ^2^ Rheumatology Clinic, Sahlgrenska University Hospital, Gothenburg, Sweden; ^3^ Department of Clinical Immunology and Transfusion Medicine, Sahlgrenska University Hospital, Gothenburg, Sweden

**Keywords:** Rho-GTPases, CD14^+^ cells, synovia, arthritis, oxidative phosphorylation, proteasome

## Abstract

**Objective:**

Activation of Rho-GTPases in macrophages causes inflammation and severe arthritis in mice. In this study, we explore if Rho-GTPases define the joint destination of pathogenic leukocytes, the mechanism by which they perpetuate rheumatoid arthritis (RA), and how JAK inhibition mitigates these effects.

**Methods:**

CD14^+^ cells of 136 RA patients were characterized by RNA sequencing and cytokine measurement to identify biological processes and transcriptional regulators specific for *CDC42* ^hi^CD14^+^ cells, which were summarized in a metabolic signature (MetSig). The effect of hypoxia and IFN-γ signaling on the metabolic signature of CD14^+^ cells was assessed experimentally. To investigate its connection with joint inflammation, the signature was translated into the single-cell characteristics of *CDC42* ^hi^ synovial tissue macrophages. The sensitivity of MetSig to the RA disease activity and the treatment effect were assessed experimentally and clinically.

**Results:**

*CDC42* ^hi^CD14^+^ cells carried MetSig of genes functional in the oxidative phosphorylation and proteasome-dependent cell remodeling, which correlated with the cytokine-rich migratory phenotype and antigen-presenting capacity of these cells. Integration of *CDC42* ^hi^CD14^+^ and synovial macrophages marked with MetSig revealed the important role of the interferon-rich environment and immunoproteasome expression in the homeostasis of these pathogenic macrophages. The *CDC42* ^hi^CD14^+^ cells were targeted by JAK inhibitors and responded with the downregulation of immunoproteasome and MHC-II molecules, which disintegrated the immunological synapse, reduced cytokine production, and alleviated arthritis.

**Conclusion:**

This study shows that the CDC42-related MetSig identifies the antigen-presenting CD14^+^ cells that migrate to joints to coordinate autoimmunity. The accumulation of *CDC42* ^hi^CD14^+^ cells discloses patients perceptive to the JAKi treatment.

## 1 Introduction

Monocytes are the crucial innate effectors in the pathogenesis of the canonical inflammatory joint disease rheumatoid arthritis (RA). Monocytes are a heterogenous population that, in healthy individuals, maintains the immune homeostasis of joint tissues supporting renewal and anti-microbial protection (1, 2). The migration and infiltration of pro-inflammatory monocytes into synovial tissue is an early sign of RA (3–5). Pro-inflammatory monocytes differentiate to macrophages and play a key role in the propagation of synovial inflammation by maintaining a continuous influx of leukocytes into the joint compartment of RA patients (3).

Monocytes, together with fibroblast-like synoviocytes, present the main pool of antigen-presenting cells in the joint cavity (6, 7). During inflammation, CD14^+^ monocytes increase in the circulation of RA patients and are characterized by a high oxygen consumption rate and the number of mitochondria (8). In contrast to healthy individuals, the inflamed synovium is hypoxic (9, 10), which transforms the monocyte subsets with respect to the surface receptor phenotype, antigen-presenting capacity, and cytokine production.

The key in the antigen presentation process is the generation of peptides for loading onto the major histocompatibility complex (MHC) for presentation to CD4^+^ and CD8^+^ T cells. Although the fragmentation of proteins in the proteasome complex followed by loading within the endoplasmic reticulum has initially been identified in MHC class I receptors (MHC-I) interacting with CD8^+^ (11), this process appeared to be equally relevant for MHC class II receptors (MHC-II) and CD4^+^ cells (12). The proteasome complex is an integral part of cell homeostasis. The constitutive 26S proteasome consists of a barrel-shaped catalytic core and a regulatory lid associated to it. To be disintegrated by constitutive proteasome, the protein needs to be ubiquitinated, unfolded by the regulatory lid, and catalyzed into peptides. All these events are dependent on the energy released by the hydrolysis of ATP (13, 14). To generate peptides suitable for the antigen presentation by MHC complexes, the proteasome changes its composition. Specifically, the regulatory lid is replaced by an open-gate proteasome activator complex PA28 encoded by *PSME1*, *PSME2*, and *PSME3*, and the catalytic core replaces three of its catalytic β-subunits encoded by *PSMB6*, *PSMB7*, and *PSMB5* with different endopeptidases encoded by *PSMB9*, *PSMB10*, and *PSMB8*, respectively. These changes recognize the immunoproteasome complex, which has ATP-independent dynamics of protein degradation and results in the generation of more hydrophobic peptides (15). Several conditions induce a transition of the constitutive proteasome to immunoproteasome. Among those are exposure to pro-inflammatory cytokine interferons (IFN-α, IFN-β, and IFN-γ), TNF-α, and stress factors, including oxygen deprivation, exposure to toxins, etc. (16).

In our previous studies, we found that the conditional knockout of GGTase-I protein (GLC) in mouse macrophages caused a symmetrical arthritis and skeletal damage in small joints, which was morphologically identical to human RA. The GLC macrophages were recognized by an accumulation of the activated forms of RAC1, RHOA, and CDC42 GTPases (17, 18). Rho-GTPases are signal transducers that regulate the actin cytoskeleton and cell migration but have recently been implicated in vital cellular functions of metabolic regulation, differentiation, and cell cycle control (19, 20). Additionally, Rho-GTPases are engaged in the process of antigen presentation—for example, it has been shown that constitutively active CDC42 mediates antigen presentation between dendritic cells and T cells (21), while *CDC42* deficiency led to a reduction in lysosomal content and upregulation of dysfunctional invariant chain (22). Concordantly, we demonstrated that GLC macrophages efficiently transduced the upregulation of Rho-GTPases to CD4^+^T cells and created an IFN-γ rich environment. This resulted in an excessive egress of regulatory T cells to the periphery that later acquired an invasive phenotype and migrated into the joints, thus causing arthritis (23). Similar to the *GLC* macrophages, the deletion of *Pggt1b* in CD4^+^ cells resulted in hyperactivated Rho-GTPases and aggravated autoimmune colitis (24). The modulation of Rho-GTPases in experimental autoimmunity efficiently changed the disease severity. The deletion of the activated Rac1 and RhoA in GLC macrophages resulted in the alleviation of arthritis in mice (23, 25). Consistently, RhoA-deficiency in CD4^+^ cells alleviated autoimmune encephalomyelitis (26), while the gain-of-function mutation in the *RHOA* gene induced a T cell-dependent autoimmunity (27). The conditional deletion of CDC42 demonstrated that this Rho-GTPase mediated an interaction between macrophages and CD4^+^ cells, which promoted T cell expansion and invasion (23, 28, 29).

**Figure 1:**
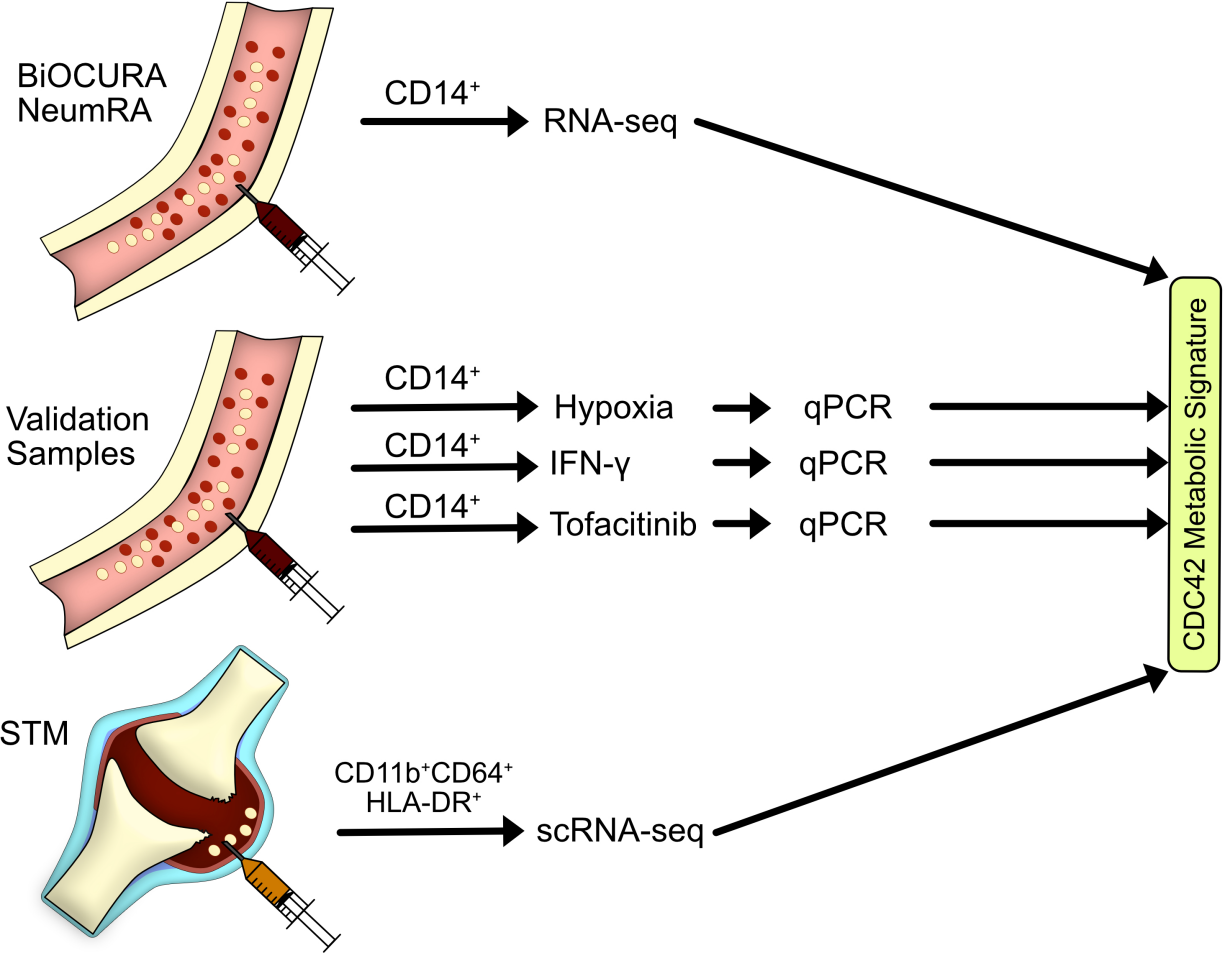
Flow chart of materials and sample analysis. In this study, we explored the transcriptome of blood CD14^+^ cells of rheumatoid arthritis (RA) patients in the BiOCURA and NeumRA cohorts and identified a CDC42-related metabolic signature in those cells. Experimental validation of the metabolic signature was done on blood CD14^+^ cells of healthy individuals subjected to hypoxia, IFN-γ, and JAK inhibitor. An investigation of RA synovial tissue macrophages defined as CD11b^+^CD64^+^HLA-DR^+^ cells demonstrated that the antigen-presenting cells marked by the CDC42-related metabolic signature migrate to RA joints to coordinate autoimmunity. This population of CD14^+^ cells was sensitive to JAK inhibition.

The findings in experimental arthritis set the starting point of this study, in which we translate the Rho-GTPase dependent mechanism of arthritis into the metabolic signature of *CDC42^hi^* CD14^+^ cells of RA patients and investigate the role of these cells in the perpetuation of synovial inflammation. Integrating independent sample collections and single-cell resolution transcriptomics, we explored the process of *CDC42^hi^* CD14^+^ cell transformation in the synovium with a focus on antigen presentation and proteasome-dependent protein remodeling (**Figure 1**). We demonstrated the key role of CDC42-dependent mechanisms in the pathogenic migration of macrophages into synovial tissue during RA. Finally, we examined the ability of anti-rheumatic treatments to affect these mechanisms and exposed their reversible nature.

## 2 Materials and methods

### 2.1 Patients

Blood samples of 59 female RA patients were collected at the Rheumatology Clinic, Sahlgrenska Hospital, Gothenburg. The clinical characteristics of the patients are shown in **Supplementary Table S1**. All RA patients fulfilled the EULAR/ACR classification criteria (30) and gave a written informed consent before the blood sampling. The study was approved by the Swedish Ethical Review Authority (659–2011) and done in accordance with the Declaration of Helsinki. The trial is registered at ClinicalTrials.gov (ID NCT03449589). In this study, CD14^+^ and CD4^+^ cells from RA patients were used for RNA-seq, qPCR, ELISA, and culturing experiments in a hypoxic environment with and without IFN-γ stimulation as well as culturing experiments with JAK inhibitors. An additional RNA-seq dataset of CD14^+^ cells from 77 RA patients with active disease and naïve to treatment was accessed (accession no. GSE138747) (31) from the Gene Expression Omnibus database (GEO, RRID : SCR_005012). The corresponding clinical data of these 77 patients was kindly provided by Dr. Tao and is shown in **Supplementary Table S1**. Lastly, the scRNA-seq dataset of synovial tissue HLA-DR^+^CD11b^+^ macrophages sorted by flow cytometry from the arthroscopic biopsies of 25 RA patients (32) was accessed from the EMBL’s European Bioinformatic Institute depository (accession no. E-MTAB-8322, RRID : SCR_004727).

### 2.2 Isolation of CD14^+^ and CD4^+^ cells

Human peripheral blood mononuclear cells were isolated from venous peripheral blood by density gradient separation on Lymphoprep (Axis-Shield PoC As, Dundee, Scotland). CD4^+^ cells were isolated by positive selection (11331D; Invitrogen, Waltham, MA, USA) and cultured (1.25 × 10^6^ cells/mL) in wells coated with anti-CD3 antibody (0.5 mg/mL; OKT3, Sigma-Aldrich, Saint Louis, MO, USA, RRID : AB_2619696) in RPMI medium supplemented with 50 µM β2-mercaptoethanol (Gibco, Waltham, MA, USA), 2 mM Glutamax (Gibco), 50 µg/mL Gentamicin (Sanofi-Aventis, Paris, France), and 10% fetal bovine serum (Sigma-Aldrich) at 37°C in a humidified 5% CO_2_ atmosphere for 48 h. CD14^+^ were subsequently purified from the remaining cell mixture by positive selection (17858; Stem Cell Technologies, Vancouver, Canada) and cultured in the same medium and conditions as the CD4^+^ cells but stimulated with lipopolysaccharide (LPS; 5 µg/mL; Sigma-Aldrich). Culture supernatants were collected for the analysis of cell products by ELISA.

### 2.3 Culturing experimental CD14^+^ cells

Human peripheral blood mononuclear cells were isolated from venous peripheral blood by density gradient separation on Lymphoprep (Axis-Shield PoC As). CD14^+^ cells were isolated by positive selection (Stem Cell Technologies) and cultured (1.25 × 10^6^ cells/mL) in RPMI medium supplemented with 50 µM β2-mercaptoethanol (Gibco), 2 mM Glutamax (Gibco), 50 µg/mL Gentamicin (Sanofi-Aventis), and 10% fetal bovine serum (Sigma-Aldrich). In the experiment, determining the effect of hypoxia and IFN-γ cell stimulation was performed by the addition of LPS (5 µg/mL; Sigma-Aldrich) and IFN-γ (50 ng/mL; Peprotech, Cranbury, NJ, USA) in either normoxic (95% air, 5% CO_2_) or hypoxic (1% O_2_, 5% CO_2_, and 94% N_2_) conditions over 48 h at 37°C. To determine the effect of JAK inhibitors, the cells were stimulated by the addition of LPS (5 µg/mL; Sigma-Aldrich) with or without tofacitinib (10 µM; CP-690550; Selleck Chemicals, Houston, TX, USA).

### 2.4 Transcriptional sequencing (RNA-seq)

RNA was prepared with the Norgen Total RNA purification kit (37500; Norgen Biotek, Ontario, Canada). Quality control was done with a Bioanalyzer RNA6000 Pico on Agilent2100 (Agilent, St. Clara, CA, USA, RRID : SCR_019715). Deep sequencing was done by RNA-seq (Hiseq2000; Illumina, San Diego, CA, USA, RRID : SCR_020132) at the LifeScience Laboratory, Huddinge, Sweden. Raw sequence data were obtained in Bcl files and converted to fastq text format with bcl2fastq. The RNA-seq results were validated by qRT-PCR as described below. The Fastq-files and the processed reads are deposited in Gene Expression Omnibus at the National Centre for Biotechnology Information with the accession number GSE201669.

### 2.5 Conventional qPCR

RNA was isolated with Total RNA Purification Kit (37500; Norgen Biotek). The RNA concentration and quality were evaluated with a NanoDrop spectrophotometer (Thermo Fisher Scientific, RRID : SCR_018042) and Experion electrophoresis system (Bio-Rad Laboratories, RRID : SCR_019691). cDNA was synthesized from RNA (200 ng) with the High-Capacity cDNA Reverse Transcription Kit (4368814; Applied Biosystems, Foster City, CA, USA). Real-time amplification was done with RT^2^ SYBR Green qPCR Mastermix (330522; Qiagen, Hilden, Germany) and ViiA 7 Real-Time PCR System (Applied Biosystems, RRID : SCR_023358) as described (33). The melting curves for each PCR were performed between 60°C and 95°C to ensure the specificity of the amplified product. All samples were run in duplicate with ACTB (beta-actin) as a reference gene and with a negative control. The expression levels of target genes were normalized to ACTB to obtain the difference in cycle threshold (dCt) using the QuantStudio™ Real-time PCR software (v1.3; Applied Biosystems). The relative quantity was calculated using the ddCt method. The primers used are shown in **Supplementary Table S2**.

### 2.6 ELISA cytokine measurement

The cytokine levels were measured with a sandwich enzyme-linked immune assay (ELISA) as below. Briefly, high-performance 384-well plates (Corning Plasticware, Corning, NY, USA) were coated with capture antibody, blocked, and developed according to the manufacturers’ instructions. The developed plates were read by a SpectraMax340 Microplate reader (Molecular Devices, San Jose, CA, USA, RRID : SCR_020303) at the dual wavelength of 450/650 nm, and the absolute protein levels were calculated after serial dilutions of the recombinant protein provided by the manufacturer. The following reagents were used: for IFN-γ (detection limit: 3 pg/mL, PelikineM1933, Sanquin, Amsterdam, The Netherlands, RRID : AB_2935684), TNF-α (detection limit: 15.6 pg/mL, DY210, R&D Systems, RRID : AB_2848160), IL-1β (detection limit: 3.9 pg/mL, DY201, R&D Systems, RRID : AB_2848158), IL-6 (detection limit: 9.4 pg/mL, DY206, R&D Systems, RRID : AB_2814717), CXCL8 (detection limit: 31.2 pg/mL, DY208, R&D Systems), and IL-10 (detection limit: 15 pg/mL, DY217B, R&D Systems, RRID : AB_2927688).

### 2.7 RNA-seq analysis

Transcripts were mapped with the UCSC Genome Browser using the annotation set for the hg38 human genome assembly and analyzed with the core Bioconductor packages in RStudio (v 4.1.1, RRID : SCR_000432). DEGs were identified with DESeq2 (v 1.26.0, RRID : SCR_015687) (34). Genes were considered to be differentially expressed if *p*_nominal_ *<*0.05. ComplexHeatmap (v 2.8.0, RRID : SCR_017270) was used to cluster genes and construct heatmaps. Pathway enrichment analysis was performed using g:Profiler web client (ELIXIR Infrastructure, RRID : SCR_006809) (35), with a term size filter of 1000. Enrichment of transcription factor targets were determined using the GSEA web client (Broad Institute, RRID : SCR_016863) (36), testing genes against the TFT collection (GTRD (37) and LEGACY).

### 2.8 Single-cell RNA sequencing analysis

Synovial tissue macrophages of interest (STM) were identified in a scRNA-seq dataset obtained by Alivernini, MacDonald (32). Utilizing Seurat R package (v 4.1.0, RRID : SCR_016341) (38), cells with unique feature counts over 4,500 or less than 500 and >20% mitochondrial counts were analyzed. The dimensionality of the dataset was determined with the ElbowPlot() function. Cells in the dataset were clustered into 15 clusters by unsupervised Uniform Manifold Approximation and Projection (UMAP). The *CDC42^hi^MetSig^hi^* STM cluster was identified by the summation of the aggregate expression of each gene in MetSig (*ATP5BP*, *COX7A2*, *PSMB6*, *PSME3*, *GTF3C6*, and *GTF2E2*), by extracting with the AggregateExpression() function and dividing by the number cells of each cluster. The FindAllMarkers() function was used to determine the markers of the *CDC42^hi^MetSig^hi^* STM cluster, using the default Wilcoxon rank sum test method with the remaining cluster as comparison. The genes were considered markers if |log_2_FC| >0.25 and with minimum feature percentage detection at 25%.

### 2.9 Regression model

A linear equation between the point representing the maximums and the point representing the minimums of the variables of interest was formulated. Using the equation, a predicted value was calculated, and samples were considered inside the model if the absolute value of the difference between the predicted and real value was less than 1.

### 2.10 Principal component analysis

Principal components analysis was performed using PCA() function of R package FactorMineR (v 2.6, RRID : SCR_014602) and visualized using the fviz_pca_biplot() function of factoextra (v 1.0.7, RRID : SCR_016692).

## 3 Results

### 3.1 Enriched oxidative phosphorylation and transcriptional regulation in *CDC42^hi^* CD14^+^ cells

**Figure 2:**
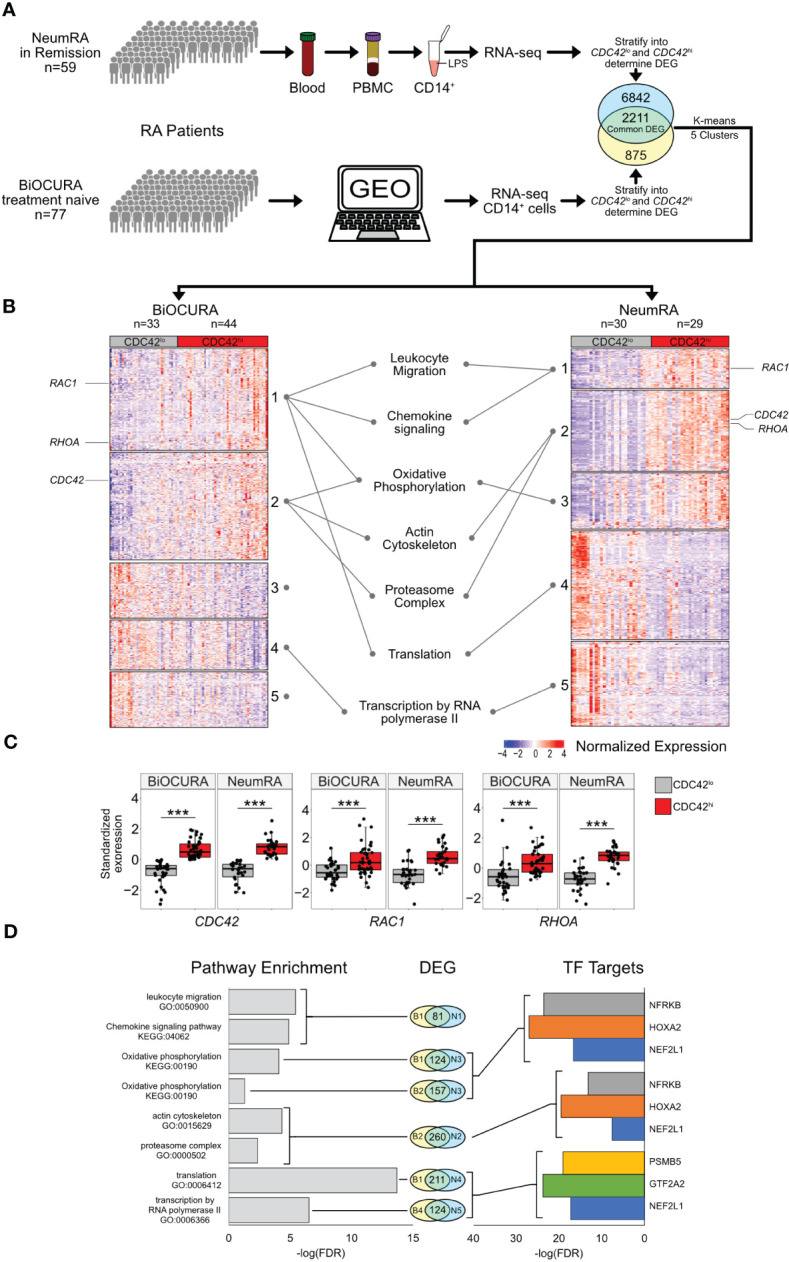
Transcriptional characteristics of *CDC42^hi^* CD14^+^ cells of two independent RA cohorts. **(A)** Whole-genome RNA was analyzed by sequencing in blood CD14^+^ cells of BiOCURA (n=77) and NeumRA cohorts (n=59). Comparison between *CDC42^hi^* and *CDC42^lo^* (split by mean) CD14^+^ cells was done by DESeq2. The identified differentially expressed genes (DEG, nominal p<0.05) were clustered by K-means. **(B)** Heatmap of DEG common for both cohorts. **(C)** Box plots of Rho-GTPase *CDC42, RHOA* and *RAC1* expression after Z-transformation. (***) indicates p-value<0.001. **(D)** Bar plots of false discovery rate (FDR) for GO:biological processes and KEGG pathways and transcription factor (TF) targets enriched within each cluster.

To investigate the complete transcriptome of *CDC42* ^hi^CD14^+^ cells, we utilized 136 RNA-seq datasets of blood CD14^+^ cells from two independent cohorts of RA patients. The cells with *CDC42* expression above the mean of each cohort (*CDC42* ^hi^CD14^+^ cells) (**Supplementary Figure S1**) were significantly enriched with other canonical Rho-GTPases *RAC1* and *RHOA* (**Figure 2C**), which indicated the phenotypic identity of CD14^+^ cells of the BiOCURA and NeumRA cohorts. The differentially expressed genes (DEG, normalized *p*-value <0.05) in *CDC42^hi^* CD14^+^ cells were compared. The analysis showed that 2,211 DEGs were common for the BiOCURA and NeumRA cohorts and comprised 71% and 24% of the complete set of DEGs within each cohort, respectively (**Figure 2A**). To enrich for similarity, we performed an unsupervised co-expression-based clustering of DEGs within each cohort. The pathway enrichment analysis of the common DEGs revealed that the *CDC42* ^hi^CD14^+^ cells were engaged in the processes of leukocyte migration (GO:0050900, FDR = 3.65e^-06^) and chemokine signaling (KEGG:04062, FDR = 1.27e^-05^), which are among the best characterized functions of Rho-GTPases (**Figure 2B**) (19). Additionally, the DEG regulated energy supply through the control of the oxidative phosphorylation (KEGG:00190, FDR = 8.18e^-05^), which supported actin cytoskeleton (GO:0015629, FDR = 4.69e^-05^) and proteasome complex function (GO:0000502, FDR = 4.38e^-03^), followed by transcription (GO:0006366, FDR = 2.91e^-07^) and translation (GO:0006412, FDR = 2.04e^-14^) (**Figure 2D**). This illustrated the ability of Rho-GTPases to orchestrate a chain of cell functional changes.

With focus on these processes, we performed a search for the upstream transcriptional regulators of the DEGs within individual clusters using the TF target collection in the Molecular Signature Database (MSigDB, GSEA, Broad Institute). Consistent with the results of the pathway analysis, we observed an enrichment for the gene targets of nuclear factor erythroid 2-related factor 1 (NFE2L1, *n* = 283), homeobox A2 (HOXA2, *n* = 219), and nuclear factor related to Kappa-B binding protein (NFRKB, *n* = 228) TF families (**Figure 2D**). These TFs together regulated oxidative phosphorylation (OXPHOS) through an antioxidant response motif and DNA remodeling and repair (39–41) during the actin cytoskeleton and proteasome complex function (**Figure 2D**).

**Figure 3:**
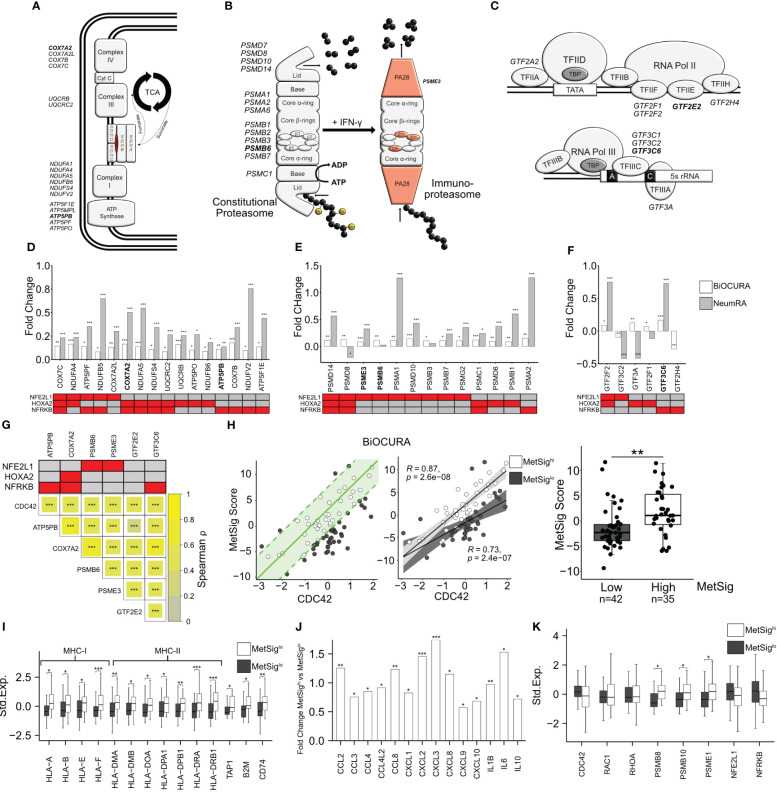
CDC42-related metabolic signature of CD14^+^ cells. **(A)** Electron transport chain complexes in the inner mitochondrial membrane and the tricarboxylic acid cycle. **(B)** Constitutive proteasome and immunoproteasome complexes. **(C)** Composition of the transcription initiation complexes. Genes differentially expressed in *CDC42^hi^* CD14^+^ cells are indicated. **(D–F)** Bar plot of the fold change transcription difference between *CDC42* ^hi^ and *CDC42* ^lo^ CD14^+^ cells. BiOCURA, white bars. NeumRA, gray bars. The transcriptional regulators for each gene are shown in red. **(G)** Heat map of Spearman correlation between the transcription of *CDC42* and the metabolic signature (MetSig) genes. The transcriptional regulators for each gene are shown in red. **(H)** Scatter plot of relationship between the MetSig and *CDC42* expression. The green line indicates the linear regression model. CD14^+^ cells within one standard deviation (green area) from the model are shown by open circles. Spearman correlation value between the MetSig and *CDC42* expression within the groups. Box plot of the MetSig for CD14^+^ cells within (open circles, MetSig^hi^) and outside (solid circles, MetSig^lo^) the regression model. *P*-value is calculated by Wilcoxon rank-sum test. ** indicates *p*-value <0.01. **(I)** Box plot of genes engaged in antigen presentation in MetSig^hi^ (white bars) and MetSig^lo^ (gray bars) CD14^+^ cells. Gene expression is shown after z-transformation. **(J)** Bar plot of gene transcription difference in fold change between MetSig^hi^ and MetSig^lo^ CD14^+^ cells. **(K)** Box plot of gene transcription in MetSig^hi^ (white bars) and MetSig^lo^ (gray bars) CD14^+^ cells. *P*-values are calculated by DESeq2 test. * indicates *p*-value <0.05. ** indicates *p*-value <0.01. *** indicates *p*-value <0.001.

Since the covariance clustering demonstrated that *CDC42^hi^* CD14^+^ cells were characterized by the activation of OXPHOS, we performed an in-depth analysis of the individual electron transport chain (ETC) complexes and the tricarboxylic acid (TCA) cycle (**Figure 3A**). We found that a substantial number of ETC genes were enriched in *CDC42^hi^* CD14^+^ cells of the BiOCURA and NeumRA cohorts. Furthermore, many of those DEG were direct targets of NFE2L1, HOXA2, and NFRKB and coded for the components of ATP synthase complex (*ATP5PO*, *ATP5PF*, *ATP5PB*, and *ATP5F1E*), NADH–ubiquinone oxidoreductase complex (Complex I, *NDUFA4*, *NDUFA5*, *NDUFB5*, *NDUFB6*, *NDUFS4*, *NDUFV2*), coenzyme Q-cytochrome c reductase complex (Complex II, *UQCRB*, *UQCRC2*), and cytochrome oxidase subunit 7 (Complex IV, *COX7A2L*, *COX7B*, *COX7C*) (**Figures 3A, D**). Additionally, the *CDC42^hi^* CD14^+^ cells had significantly upregulated NFRKB targets including the glucose transporter gene *SLC2A1*, and *SUCLG1* and SUCLG2, that catalyzes the conversion of succinyl CoA to succinate (**Supplementary Figure S2A**). NFE2L1, NFRKB, and HOXA2 control protein remodeling, including proteasomal degradation (GO:0000502, FDR = 4.83e^-03^) and RNA polymerase synthesis (GO:0006366, FDR = 2.91e^-07^) (**Figures 2D**, **3B, C**). Proteins forming the 26S proteasome were highly expressed in *CDC42^hi^* cells of both cohorts (**Figure 3E**), consistent with the enhanced protein remodeling process in those cells. This included PA28-specific subunit *PSME3*, the ATPase 1 protein coded by *PSMC1*, components of the β-ring catalytic core *PSMB6* and *PSMB7*, and components of the non-ATPase regulatory lid *PSMD7*, *PSMD8*, *PSMD10*, and *PSMD14*, many of which were under the transcriptional control of NFE2L1 (39). The general transcription factors (GTF) that mobilize the RNA polymerase complexes to chromatin were among the transcriptional targets of NFE2L1, HOXA2, and NFRKB upregulated in *CDC42^hi^* CD14^+^ cells (**Figure 3F**). Notably, the proteasome subunit PSMB5 and the GTF member GTF2A2 had an independent, often functionally paired, transcriptional control of translation through the genes coding for AP-1 TFs, ribosomal and histone proteins, as well as α- and β-tubulin that enable RNA binding activity (**Supplementary Figures S2B, C**).

Taken together, the TF target analysis demonstrated that *CDC42^hi^* CD14^+^ cells are programmed via TFs NFE2L1, HOXA2, and NFRKB to mediate the strong connection between Rho-GTPases, OXPHOS activity, and proteasome-dependent protein remodeling.

### 3.2 Metabolic signature defines the antigen presenting and migratory phenotype of *CDC42^hi^* CD14^+^ cells

Based on the knowledge gained with the analysis of the biological processes, we identified a set of internally related genes characteristic for the metabolic signature (MetSig) of *CDC42^hi^* CD14^+^ cells and representing OXPHOS and proteasome-dependent remodeling. To be included in this *CDC42*-related MetSig, the gene should be significantly upregulated in *CDC42^hi^* CD14^+^ cells, transcriptionally controlled by any of NFE2L1, HOXA2, and NFRKB TFs, and correlated with *CDC42* and other genes in the signature (**Figure 3G**). Hence, the correlation between the MetSig and *CDC42* expression was strong in CD14^+^ cells of both RA cohorts (BiOCURA, *r* = 0.65, *p* < 2e-16; NeumRA, *r* = 0.80, *p* < 2e-16, **Supplementary Figure S2D**). Concordantly, MetSig was significantly upregulated in *CDC42^hi^* CD14^+^ of both cohorts (**Supplementary Figure S2G**). To identify CD14^+^ cells carrying the CDC42-related MetSig, we constructed a linear regression model between the maximal and the minimal sum of the standardized expression of the signature genes and *CDC42* expression (**Figure 3H**). We observed that the CD14^+^ cells in the BiOCURA cohort of patients with active RA presented two independent groups, where the MetSig had a direct correlation with the *CDC42* gene (**Figure 3H**). The CD14^+^ cells within the model had higher MetSig (*MetSig^hi^*, *n* = 35) compared to those outside the model (*MetSig^lo^* cells, *n* = 42), while the expression of *CDC42* between the groups was comparable (**Supplementary Figure S2E**). We found no difference in the part of samples taken from male and female individuals between the MetSig groups. To investigate the phenotype distinctions between the *MetSig* ^hi^ and *MetSig* ^lo^ CD14^+^ cells, we analyzed the differentially expressed genes. In addition to translation, proteasome function, and OXPHOS, the genes upregulated in *MetSig^hi^* CD14^+^ cells were functional in antigen presentation (GO:0019882, FDR = 1.72e^-06^) and leukocyte chemotaxis (GO:0030595, FDR = 3.49e^-05^) (**Supplementary Figure S2F**). The antigen presentation was reflected by numerous HLA genes including the strongest known RA-risk gene *HLA-DRB1* as well as β_2_-microglobulin (*B2M)*, ATP-binding cassette transporter *TAP1*, and invariant chain *CD74/CLIP* responsible for the unidirectional translocation of antigen peptides and loading into the HLA groove for presentation to CD4^+^T cells (**Figure 3I**). The proteasome complex was enriched with the immunoproteasome subunits *PSMB8*, *PSMB10*, and *PSME1*, consistent with the acquisition of the antigen presenting function by the *MetSig^hi^* CD14^+^ cells (**Figure 3K**). The *MetSig^hi^* CD14^+^ cells produced a broad spectrum of pro-inflammatory C-C and C-X-C chemokines including CCL2, CCL3, CCL8, CXCL8, and CXCL10 as well as the cytokines IL-1β, IL-6, and IL-10 (**Figure 3J**). This abundance in chemoattractants lent support to a connection between the MetSig and the migratory phenotype of *CDC42^hi^* CD14^+^ cells and reflected the functional importance of these cells in the Rho-GTPase-dependent inflammation in RA.

### 3.3 CD14^+^ cells with high metabolic signature migrated into the synovial tissue in RA

**Figure 4:**
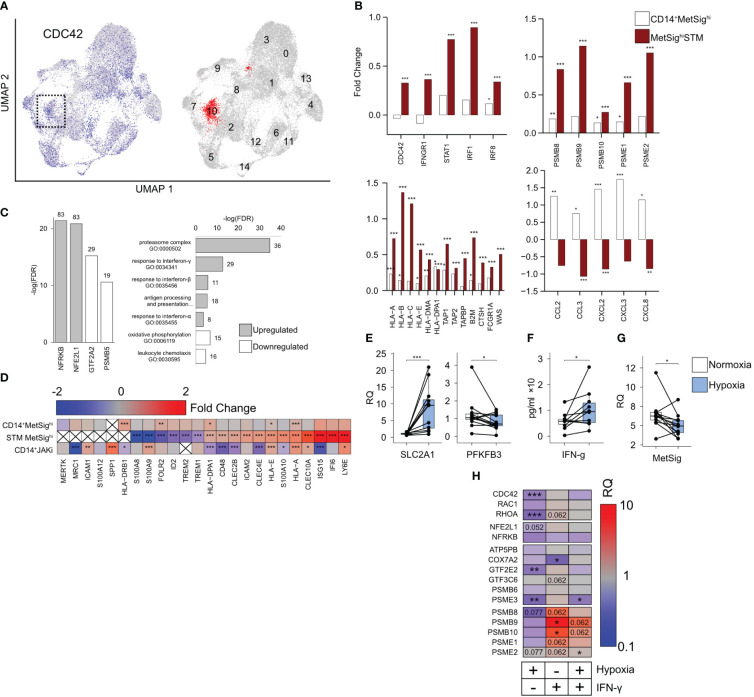
*CDC42^hi^MetSig^hi^* cluster of synovial tissue macrophages (STM). **(A)** Uniform Manifold Approximation and Projection of the single-cell RNA-seq shows the distribution of CD11b^+^CD64^+^HLA-DR^+^ cells with a high expression of *CDC42* (blue). The cluster with the highest metabolic signature (MetSig) is marked red. **(B)** Bar plot of transcription difference in fold change (FC) between MetSig^hi^ and MetSig^lo^ CD14^+^ cells (open bars) and between MetSig^hi^STM and other STM clusters (red bars). **(C)** Bar plots of the false discovery rate for the GO:biological processes and transcription factor targets enriched (gray bars) and downregulated (open bars) in the MetSig^hi^STM cluster. **(D)** Heat map of the transcription difference in FC between MetSig^hi^ and MetSig^lo^ CD14^+^ cells, MetSig^hi^STM cluster, and CD14^+^ cells of JAKi-treated patients. *P*-values are calculated by DESeq2 test for bulk RNA-seq and Wilcoxon rank-sum test for scRNA. * indicates *p*-value <0.05. ** indicates *p*-value <0.01. *** indicates *p*-value <0.001. **(E)** Box plot of *SLC2A1* and *PFKFB3* mRNA in CD14^+^ cells (*n* = 12) cultured in hypoxia (1% O_2_) for 48 h. Relative quantity (RQ) was calculated in relation to *ACTB* gene. **(F)** Box plot of IFN-γ protein levels (by ELISA) in the supernatants of CD14^+^ cells (*n* = 10) cultured in hypoxia. **(G)** Box plot of the MetSig genes expression in CD14^+^ cells (*n* = 12) cultured in hypoxia. Relative quantity was calculated in relation to the ACTB gene. **(H)** Heat map of the transcription difference in RQ between CD14^+^ cells (*n* = 12) cultured in hypoxia and IFN-γ (0 and 50 ng/mL). The transcription is normalized to control CD14^+^ cells cultured without IFN-γ in normoxic conditions. *P*-values are calculated by Wilcoxon signed-rank test. * indicates *p*-value <0.05. ** indicates *p*-value <0.01. *** indicates *p*-value <0.001.

To investigate if *CDC42^hi^* CD14^+^ cells were committed to migrate into inflamed joints in RA, we turned our attention to a recent atlas of the CD11b^+^CD64^+^HLA-DR^+^ synovial tissue macrophages (STM) at the level of single-cell resolution (32). First, we utilized the UMAP analysis to find the STM clusters with high RNA levels of *CDC42*. Applying the expression of *CDC42* and the MetSig genes, we identified the STM cluster which combined the high *CDC42* expression and high MetSig and, in this respect, had a remarkable similarity with the blood *CDC42^hi^* CD14^+^ cells (**Figure 4A**, **Supplementary Figures S3A, B**). In the next step, we compared the transcriptomics of the *CDC42^hi^* STM cluster to the remaining cells in the given scRNA-seq and extracted the unique transcriptional characteristics of this cluster. Similar to the blood *CDC42^hi^* CD14^+^ cells, the *CDC42* ^hi^STM cluster expressed Rho-GTPases *RAC2*, *RHOC*, and *RHOB*, represented the genes operated in proteasome complex and antigen presentation, and were responsive to IFN-γ. Similar to the blood *CDC42^hi^* CD14^+^ cells, the *CDC42* ^hi^STM cluster accumulated the transcriptional targets of NFE2L1 and NFRKB (**Figure 4C**). Specifically, *CDC42^hi^* STM expressed the complete set of immunoproteasome subunits *PSMB8*, *PSMB9*, *PSMB10*, and PA28 proteins *PSME1* and *PSME2* (**Figure 4B**). This immunoproteasome domination was combined with high mRNA levels of the HLA-A, HLA-E, and HLA-DPA genes and the peptide transporters *TAP1*, *TAP2*, *TAPBP*, and *FCGR1A* (coding for CD64) which load the peptides into the MHC complex as well as the actin-nucleation-promoting factor WASP (encoded by *WAS*), an effector of CDC42, which enriched the filopodial protrusions with MHC receptors (**Figure 4B**).

The top upregulated genes in the *CDC42^hi^* STM cluster were the IFN-γ-sensitive genes *ISG15* and *IFI6* and *Ly6E* (**Figure 4D**), which revealed that *CDC42^hi^* STM represented IFN-γ-activated cells with a consecutive expression of the *IFNGR1*, *STAT1*, *IRF1*, and *IRF8* genes mediating the IFN-γ signal and numerous IFN-γ target genes including *CDC42* (**Supplementary Figure S3F**). The combination of IFN-sensitive genes with a high expression of C-type lectin receptors *CLEC10A*, *CLEC2B*, and *CLEC4E* and a low expression of the *FOLR2*, *ID2*, and *TREM1* genes typical for the resident STM (**Figure 4D**) presented a highly inflammatory subset of STM operative in autoimmune inflammation and immune cell communication (32, 42–44). Analyzing the downregulated genes, we found enrichment for the GTF2A2 and PSMB5 transcription targets as well as for the biological processes of translation and leukocyte chemotaxis (**Figure 4C**). *CDC42^hi^* STM had a low expression of CCL2, CCL3, CXCL2, CXCL3, and CXCL8 cytokines, which was opposite to the blood *MetSig^hi^CDC42^hi^* CD14^+^ cells. The comparison of blood CD14^+^ cells and STM marked by the MetSig together argued in favor of the functional transition of the *CDC42^hi^* cells from the migration-supporting chemokine production to the antigen presentation commitment after reaching the synovium.

### 3.4 Effect of oxygen deprivation and IFN-γ exposure on the metabolic signature of CD14^+^ cells

The inflamed RA synovium is hypoxic (9, 10). Consistent with oxygen deprivation, we found the downregulation of the mitochondrial gene expression in the *CDC42^hi^* STM and a decrease in the OXPHOS (**Supplementary Figure S3C**). Thus, we asked if the oxygen deprivation was responsible for the observed transition from the constitutive proteasome to the immunoproteasome. To investigate this, we cultured CD14^+^ cells under normoxic (21% O_2_) and hypoxic (1% O_2_) conditions. Hypoxia induced an expected increase of *SLC2A1* mRNA coding for the glucose transporter GLUT1, downregulated the phosphofructokinase gene *PFKFB3* (**Figure 4E**) and caused an increase in IFN-γ production by the cultured CD14^+^ cells (**Figure 4F**). Consistent with a decrease in OXPHOS, the expression of *NFE2L1*, *PSMB5*, and *GTF2A2* TFs was suppressed in hypoxic CD14^+^ cells (**Figure 4H**, **Supplementary Figure S3E**), which was followed by a significant suppression of MetSig (**Figure 4G**) and *CDC42* and *RhoA* GTPases (**Figure 4H**). However, the short-term hypoxia was not sufficient to significantly change the expression of immunoproteasome subunits in CD14^+^ cells (**Figure 4H**). In contrast, the stimulation of CD14^+^ cells with IFN-γ in normoxic conditions significantly increased the expression of the immunoproteasome genes *PSMB9* and *PSMB10*, while *PSMB8*, *PSME1*, and *PSME2* tended to decrease (**Figure 4H**). A similar effect of IFN-γ stimulation, but less pronounced, was seen in CD14^+^ cells cultured under hypoxic conditions.

These results together presented the experimental evidence that oxygen deprivation created the IFN-γ-rich environment and suppressed the *CDC42*-related MetSig in CD14^+^ cells. The IFN-γ rich conditions were required to induce immunoproteasome expression. In view of the similarity between *CDC42* ^hi^CD14^+^ and *CDC42* ^hi^STM, these findings suggest that *CDC42* ^hi^ cells bearing the MetSig were recent invaders into the synovia, not completely adapted to hypoxia.

### 3.5 *CDC42*-related metabolic signature of CD14^+^ cells was associated with RA disease severity and was modulated by the inhibition of JAK-STAT signaling

**Figure 5:**
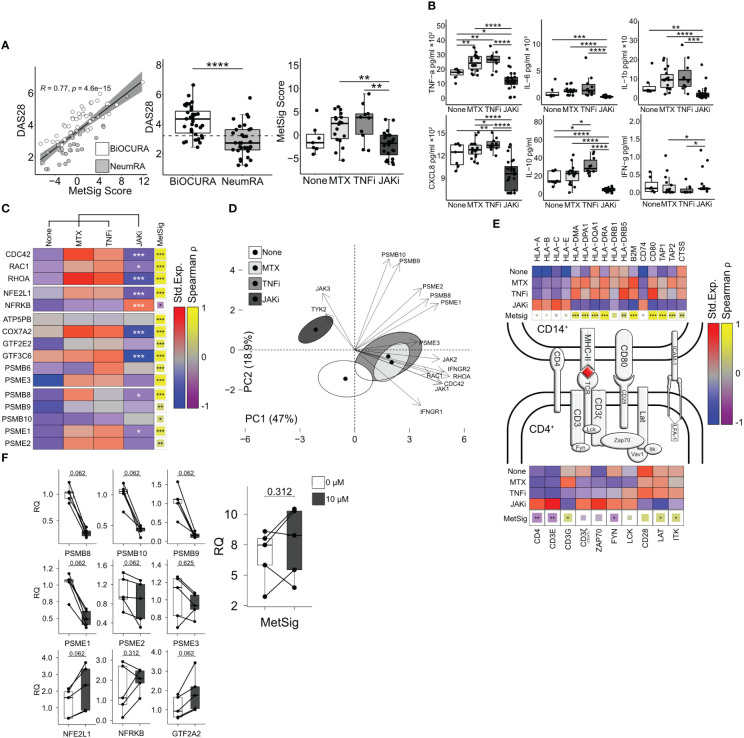
Inhibition of JAK-STAT signaling suppresses immunoproteasome and antigen presentation in CD14^+^ cells. **(A)** Dot plot of the correlation between the metabolic signature (MetSig) of CD14^+^ cells and disease activity score (DAS28). Box plot of DAS28 and MetSig in CD14^+^ cells of patients treated with methotrexate (MTX, *n* = 18), TNF inhibitors (TNFi, *n* = 10), and JAK inhibitors (JAKi, *n* = 24) and those who were not (*n* = 7). **(B)** Box plots of cytokine production (by ELISA) in CD14^+^ cells activated with lipopolysaccharide (5 µg/mL) for 48 h. *P*-values are calculated with the Mann–Whitney test. * indicates *p*-value <0.05. ** indicates *p*-value <0.01. **** indicates *p*-value <0.0001. **(C)** Heat map of the median expression after z-transformation in CD14^+^ cells. *P*-values are calculated between JAKi and other treatments by DESeq2 test. * indicates *p*-value <0.05. ** indicates *p*-value <0.01. *** indicates *p*-value <0.001. The correlation between individual genes and MetSig is calculated by Spearman correlation test. * indicates *p*-value <0.05. ** indicates *p*-value <0.01. *** indicates *p*-value <0.001. **(D)** Principal component analysis of immunoproteasome genes, IFN-γ, and Rho-GTPases in treatment groups. **(E)** Immunological synapse. Heat map of the z-normalized median expression in CD14^+^ cells and CD4^+^T cells. Correlation of gene expression to MetSig by Spearman correlation test. **(F)** Box plot of transcription change in CD14^+^ cells treated with tofacitinib (0 and 10 µM). *P*-values are calculated by Wilcoxon signed-rank test.

To investigate how the phenotype of *CDC42^hi^* CD14^+^ cells was applied to the activity of RA disease, we explored a linear regression between the MetSig of CD14^+^ cells and the disease activity score (DAS28) using it as a dependent parameter (**Supplementary Figure S4A**). In total, 52.2% of the patients (39 of 77, BiOCURA cohort; 32 of 59, NeumRA cohort) showed a strong correlation between the MetSig and DAS28 (*r* = 0.77, *p* = 4.6e^-15^. **Figure 5A**). In agreement with the nature of the cohorts, the *CDC42* ^hi^CD14^+^ cells of the BiOCURA patients with active disease had a significantly higher DAS28 (**Figure 5A**) and MetSig compared to the NeumRA cohort of treated patients. Analyzing the DEG in *CDC42^hi^* CD14^+^ cells that contributed to the RA disease activity, we identified 169 genes that correlated to DAS28 (|Spearman *ρ*| >0.5, 130 directly and 39 inversely) and confirmed their engagement in the processes of monocyte chemotaxis and proteasome complex (**Supplementary Figure S4B**). Among those, 97 (57.4%) genes were the transcriptional targets of NFE2L1, HOXA2, and NFRKB (**Supplementary Figure S4C**), adding an important link between those TFs and the perpetuation of RA disease activity.

Using the NeumRA cohort, we analyzed how RA treatment affected the CDC42-related MetSig. Overall, the CD14^+^ cells of patients treated with JAK inhibitors (JAKi, *n* = 24) had significantly lowered the MetSig compared to the CD14^+^ cells of patients treated with TNF-inhibitors (TNFi, *n* = 10) and methotrexate (*n* = 18) or not treated with anti-rheumatic drugs (*n* = 7) (**Figure 5A**). The MetSig in the treatment groups was mirrored by the expression of *CDC42*, *RAC1*, and *RHOA*, transcription factor *NFE2L1* and the immunoproteasome genes in RNA-seq (**Figure 5C**), and the protein release of IL-6, TNF-α, IL-1β, and CXCL8 by cultured CD14^+^ cells (**Figure 5B**). Principal component analysis (PCA) visualized the divergence between the CD14^+^ cells of the treatment groups regarding Rho-GTPase expression, immunoproteasome complex, and response to IFN-γ (**Figure 5D**). The first two PCs accounted for 65.9% of the total variability. PC1 (47% of variability) was mainly explained by the Rho-GTPases and IFN response genes represented by the top contributors *CDC42* and *RHOA IFNGR2* (**Supplementary Figure S4H**). PC2 (18.9% of variability) involved the immunoproteasome genes with the top contributors *PSMB9*, *PSMB10*, and *PSME2* (**Supplementary Figure S4H**).

To combine these findings with the results obtained in active RA of the BiOCURA cohort, we investigated the genes involved in antigen processing and presentation. We found that the CD14^+^ cells of the patients treated with JAKi had a low expression of MHC-II genes *HLA-DRB1* and *HLA-DPA1* and upregulated MHC-I genes *HLA-A* and *HLA-E* compared to those treated with methotrexate monotherapy or in combination with TNFi. In addition, the JAKi treatment suppressed the peptide transporters *TAP1* and *TAP2* loading on MHCI and the cathepsin S protease gene *CTSS* loading on MHC-II and upregulated the invariant chain gene *CD74*. The genes suppressed in the CD14^+^ cells of the JAKi-treated patients correlated with the MetSig (**Figure 5E**), which further confirmed a consistent effect of JAKi on the MetSig and lent us to believe that treatment with JAKi led to a restoration of healthy synovial homeostasis. Together with the reduction in Rho-GTPases and the MetSig, the CD14^+^ cells of the JAKi-treated patients had a significantly low expression of IFN-γ-dependent MHC co-receptors *CD48*, *MRC1*, and also *CLEC2B*, *CLEC4E*, and *ISG15*, while the expression of *ICAM1*, alarmin *S100A9*, *CLEC10A*, and osteopontin gene *SPP1* remained high (**Figure 4D**).

Antigen presentation through MHC class II mediates the interaction between CD14^+^ and CD4^+^ T-helper cells. Hence, we examined the genes active in the TCR complex in CD4^+^ cells which have been isolated simultaneously with CD14^+^ cells (**Figure 5E**). The analysis revealed that JAKi treatment resulted in the upregulation of the integral components of the TCR complex *CD4*, CD3ζ (encoded by *CD247*), *ZAP70*, *LCK*, *CD3E*, *FYN*, *VAV1*, and the LFA-1 subunit *ITGB2* required for TCR activation, while the co-stimulation genes *CD28*, *LAT*, and *ITK* were significantly downregulated. Accordingly, *CD4*, *CD3E*, and *FYN* were inversely correlated to the metabolic signature of CD14^+^ cells (**Figure 5E**). These findings demonstrated that treatment with JAKi caused a pronounced disbalance in the expression of the genes mediating the interaction between CD14^+^ and CD4^+^ cells, which affected the function of the immunologic synapse. This could explain the low cytokine production in the CD14^+^ cells of patients treated with JAKi (**Figure 5E**), which is dependent on those cell interactions (45, 46).

To explore if JAKi has a direct effect on the MetSig and immunoproteasome production of CD14^+^ cells, we cultured freshly isolated CD14^+^ cells in the presence of JAKi tofacitinib and measured the expression of the immunoproteasome-specific genes and the MetSig genes. We found that the CD14^+^ cells cultured with JAKi upregulated the expression of the TFs *NFE2L1* and *GTF2A2* that controlled the MetSig and downregulated the expression of the immunoproteasome subunits *PSMB8*, *PSMB9*, *PSMB10*, *PSME1*, and *PSME2* (**Figure 5F**). The MetSig of JAKi-treated cultures had no significant expression change. This indicated that the JAKi inhibitors had a direct NFE2L1-dependent effect on the immunoproteasome transcription in *CDC42^hi^* CD14^+^ cells. The observed effect was independent of CD4^+^ cells but required for optimal interaction within the immunologic synapsis.

## 4 Discussion

This study shows that circulating *CDC42^hi^* CD14^+^ cells are characterized by a specific MetSig, which combines the processes of activated oxidative phosphorylation and proteasome-dependent protein remodeling in those cells. In blood CD14^+^ cells of RA patients, the MetSig included the expression of *ATP5BP*, *COX7A2*, *PSMB6*, *PSME3*, *GTF3C6*, and *GTF2E2* genes, which individually correlated to *CDC42*, and together identified the patients where clinical RA disease activity was dependent on the CDC42-related MetSig of CD14^+^ cells. We observed that the high MetSig in *CDC42^hi^* CD14^+^ cells was associated with antigen presentation, and this phenotype was strengthened upon synovial entry in *CDC42^hi^* STM. Indeed these cells expressed multiple MHC molecules, which were accompanied by their co-receptor β_2_-microglobulin and antigen-associated invariant chain CD74/CLIP and the antigen-loading proteins TAP1 and TAP2. The *CDC42* ^hi^CD14^+^ cells marked by the high MetSig produced pro-inflammatory cytokines and a broad spectrum of chemokines, presenting a pattern of signal molecules that typically recruits immune cells to the inflamed joints in RA (2, 47). In addition to being the strongest RA risk factor, MHC molecules distinguished *CDC42* ^hi^CD14^+^ cells with efficient antigen presenting ability that accumulate the signals and mediate them further, thus coordinating the activity of the innate and adaptive immunity at the site of inflammation.

This study identified the *CDC42* ^hi^CD14^+^ cells to be ancestors of the tissue-infiltrating *CDC42* ^hi^STM, which were phenotypically united by the MetSig, the expression of MHC receptors, and the proteasome-dependent protein remodeling. Consistent with the recent analysis of the HLA-DR^+^ STM (48), the *CDC42* ^hi^STM was characterized by a suppression of the tissue-resident macrophage markers *FLOR2*, *ID2*, and *TREM2*, while the IFN-sensitive genes *ISG15*, *CLEC10A*, and *CD47* were highly expressed and revealed an invasive nature of the *CDC42* ^hi^STM, ready to maintain active synovial inflammation. The antigen presentation by MHC was combined with a high-affinity Fc-γ receptor *FCGR1A* and C-type lectin receptors to coordinate Rho-GTPase-dependent antigen uptake (49, 50). These changes together attracted the adaptive immune responses into the RA synovium. A link between high C-lectin expression in CD14^+^ cells and autoimmunity has been shown in the clinical setting and experimentally for RA (51) and multiple sclerosis (42).

Proteasome has emerged as a crucial regulator of macrophage plasticity that maintained functional proteostasis in challenged cells and tissues (52–54). Conversion into immunoproteasome helps a cell handle the excess of damaged proteins by increasing the substrate turnover capacity (55). In *CDC42* ^hi^CD14^+^ cells, we observed a general increase of the proteasome complex proteins, which was fortified by a significant enrichment with immunoproteasome in *CDC42* ^hi^STM. The turnover of Rho-GTPases in the activated cells is processed through proteasome (56). Thus, constitutive activation of Rho-GTPases in *CDC42* ^hi^ CD14^+^ cells and STM of RA patients could be a mechanism triggering immunoproteasome domination similar to that described during an infection (57, 58).

Synovial hypoxia and mitochondrial dysfunction are among the known inducers of immuno-proteasome (59) that maintain arthritis (60–62). Our findings connect the induction of immunoproteasome with the upregulation of MHCII receptors and acquisition of an immunogenic cell phenotype. We demonstrated that experimental hypoxia alone was not sufficient to induce the immunoproteasome conversion of *CDC42* ^hi^STM despite that it caused the downregulation of the MetSig in *CDC42* ^hi^CD14^+^ cells and increased glucose consumption and IFN-γ production. In turn, the IFN-rich environment created by hypoxia stimulated the production of immunoproteasome. Thus, after synovial entry, *CDC42* ^hi^CD14^+^ cells were transformed into mediators of immunological information proficient to shape the biological processes in RA synovium as we had reported in the GLC mice (18, 23).

In *CDC42^hi^* CD14^+^ cells of RA patients, OXPHOS was controlled by NFE2L1 and NFRKB TFs and supplied these cells with the energy required for chemokine-guided migration. NFE2L1 and its paralog NFE2L3 maintain the basal proteasome activity, while the simultaneous deletion of those proteins impairs proteasome function in cancer (39, 63). Acting through the downregulation of NFE2L1, autoimmune inflammation induces the proteasome subunit displacement forming the immunoproteasome (64). In this study, we found that the experimental hypoxia suppressed the mRNA levels of *NFE2L1* and let IFNγ activate the transcription of the proteasome subunits in CD14^+^ cells, while the exposure to JAKi upregulated *NFE2L1*, which decreased immunoproteasome production. Despite the fact that we found no significant difference in *NFE2L1* and *NFRKB* in *CDC42* ^hi^STM and after IFN-γ stimulation, these TFs presented a plausible checkpoint that controlled the immunoproteasome enrichment in *CDC42* ^hi^CD14^+^ cells and promoted it under the conditions of chronic IFN-γ stimulation in RA synovial tissue.

This study shows that the IFN-rich environment is a critical parameter of the *CDC42* ^hi^STM phenotype. Consistently, the abrogated IFN signal with JAKi treatment suppressed the MetSig in RA patients, which led to a dramatic change in the phenotype of CD14^+^ cells. These CD14^+^ cells downregulated the immunoproteasome genes, Rho-GTPases, and MHC-II genes, followed by the disintegration of contact with CD4^+^ cells and suppressed production of proinflammatory cytokines with a consequence of inevitably limiting synovial infiltration. The effects achieved by JAKi resembled those reported for the proteasome inhibitors in patients with autoimmune diseases and in animal models of autoimmunity (65). The success of JAKi in the treatment of the autoinflammatory syndrome caused the gain-of-function mutation in *PSMB8* and in interferonopathies, further stressing the pathogenetic closeness of these conditions (66–68). Analogously, JAKi has been shown to be efficient in controlling disease progress in multiple myeloma, where proteasome inhibitors are currently used as the first line of treatment (69).

Although exciting, the results of this study need to be challenged in a prospective setting of interventional trials using JAKi in RA and other autoimmune diseases. To disseminate the findings on the broad spectrum of available biological anti-rheumatic drugs and to confirm the predictive value of the CDC42-related MetSig for the effectiveness of other treatments, careful investigation of patients treated with abatacept, rituximab, and tocilizumab await. A recent randomized trial compared the effects of rituximab and tocilizumab in the treatment of RA (70). By deconvolution of RNA-seq from synovial biopsies, they found that the treatment response to tocilizumab was in proportion to monocyte/macrophage infiltration of the synovial tissue, which reduced with treatment. Due to the fact that the MetSig of *CDC42^hi^* CD14^+^ cells was revealed as the major migratory force of monocytes in RA, it could have mediated the effects of tocilizumab. Hence, future studies testing the MetSig of immune competent cells against targeted RA drugs may prove useful in aiming to individualize the treatment.

Taken together, this study demonstrates that *CDC42* ^hi^CD14^+^ cells are precursors of the STM cell subset with strong antigen presenting capacity that shapes and directs the adaptive immune responses in the autoimmune inflammation of RA. The study exhibits an important role of immunoproteasome in the homeostasis of such pathogenic macrophages. The CDC42-related MetSig of CD14^+^ cells identified a substantial group of RA patients in whom these molecular processes were associated and maintained the disease activity. This group of RA patients was sensitive to the treatment with JAKi and responded to the treatment with the downregulation of Rho-GTPases and immunoproteasome subunits, which resulted in a loss of interaction in the immunological synapse and in a decrease in tissue-infiltrating macrophage population. The reversible and JAKi sensitive nature of the *CDC42* ^hi^CD14^+^ cells predicts that RA patients carrying the *CDC42*-related MetSig may favor of early use of this targeted intervention, which justifies its evaluation prior to treatment choice.

## Data availability statement

The datasets presented in this study can be found in online repositories. The names of the repository/repositories and accession number(s) can be found below: https://www.ncbi.nlm.nih.gov/geo/, GSE201670, https://www.ncbi.nlm.nih.gov/geo/, GSE201669.

## Ethics statement

The studies involving humans were approved by Swedish Ethical Review Authority. The studies were conducted in accordance with the local legislation and institutional requirements. The participants provided their written informed consent to participate in this study.

## Author contributions

EM-B and MB: conceived the project and experiments. SS, RP, and MB provided samples, equipment, and reagents. EM-B, KA, and ME performed experiments and analyzed the data. EM-B and MB wrote the manuscript. All authors contributed to the article and approved the submitted version.
