# Supplementary material for: Metabolic signature and proteasome activity controls synovial migration of CDC42hi CD14+ cells in rheumatoid arthritis
Source: Front Immunol. 2023 Aug 17;14:1187093. doi: 10.3389/fimmu.2023.1187093 (PMC10469903; doi:10.3389/fimmu.2023.1187093)
Supplement: Supplementary file 1 [file DataSheet_1.docx]

Supplementary Material

Metabolic signature and proteasome activity controls synovial migration of *CDC42^hi^*CD14^+^ cells in RA

**Eric Malmhäll-Bah^1^, Karin M.E. Andersson^1^, Malin C. Erlandsson^1,2^, Sofia T. Silfverswärd, Rille Pullerits^2,3^, Maria I. Bokarewa^1,2*^**

*** Correspondence:** Maria I. Bokarewa, [maria.bokarewa@rheuma.gu.se](mailto:maria.bokarewa@rheuma.gu.se)

# Supplementary Tables

**Supplementary Table S1.** Clinical characteristics of RA patients in the BiOCURA and NeumRA cohorts.

|  | BiOCURA | NeumRA |
| --- | --- | --- |
|  | n=77 | n=59 |
| CD14 *CDC42* basemean RNAseq | 9468 | 9335 |
| Female (%) | 70.1 | 100.0 |
| Age, years (median) | 55 | 64 |
| Disease duration, years (median) | NA | 10 |
| Platelets count, 10^9^/L (median) | 285 | 257 |
| WBC count, 10^9^/L (median) | 7.6 | 5.5 |
| HB, g/L (median) | 137 | 136 |
| RF positive (%) | 67.5 | 69.5 |
| ACPA positive (%) | 74.0 | 55.9 |
| BMI, kg/m^2^ (median) | 26 | 26 |

WBC, white blood cells. HB, hemoglobin. RF, rheumatoid factor. ACPA, antibodies against cyclic citrullinated peptides. BMI, body mass index.

**Supplementary Table S2.** RT-PCR primers used in the study.

| Primer | Sequence | Tm |
| --- | --- | --- |
| *PSMB8* Forward primer | CAAGCTGCTGTCCAACATGA | 61.0 |
| *PSMB8* Reverse primer | ATTTCCTGAGAGCCGAGTCC | 60.7 |
| *PSMB9* Forward primer | CTGCTGCAAATGTGGTGAGA | 61.0 |
| *PSMB9* Reverse primer | ATTCCTCCCAGGGTTCCATA | 60.5 |
| *PSMB10* Forward primer | GTGCTAGAAGACCGGTTCCA | 60.3 |
| *PSMB10* Reverse primer | GATCACACATGCGTCCACA | 60.1 |
| *PSME1* Forward primer | GTGCCCACTCCACTCCTTGT | 62.5 |
| *PSME1* Reverse primer | GGTCTTCACGAAACACATCCA | 61.0 |
| *PSME2* Forward primer | TCCTCTACAGATTCTTGCCACAG | 60.8 |
| *PSME2* Reverse primer | GTCAGCCACATTGAGGGAGT | 60.1 |
| *PSME3* Forward primer | AAGCGAAGGTTGGATGAGTG | 60.3 |
| *PSME3* Reverse primer | AATCAACAGCCGGATCTCAG | 60.2 |
| *ATP5PB* Forward primer | CAGAACATGATGCGTCGAAA | 60.8 |
| *ATP5PB* Reverse primer | GCACTTGGCAATTGTCTCCT | 60.3 |
| *COX7A2* Forward primer | GATTGGGCAGAGGACGATAA | 60.0 |
| *COX7A2* Reverse primer | GGCTCTATACAGGAGGGCATC | 60.1 |
| *GTF2E2* Forward primer | TTGGACTCAAGCAGAAACAATG | 60.3 |
| *GTF2E2* Reverse primer | CTCACGTTGTACTTGGGCTTG | 60.7 |
| *GTF3C6* Forward primer | ATGAAGAAGCTCAGCATGACAA | 60.0 |
| *GTF3C6* Reverse primer | TCATGTTGGGTCGATAGGAGA | 60.5 |
| *PSMB6* Forward primer | GGCAGTAGCTGATGCTGTCA | 60.2 |
| *PSMB6* Reverse primer | TTAAAGAGGCTGGCTGCTGT | 60.2 |
| *RHOA* Forward primer | GTGCCCACAGTGTTTGAGAA | 59.7 |
| *RHOA* Reverse primer | GTGTCCCACAAAGCCAACTC | 60.6 |
| *RAC1* Forward primer | TGTGAGTCCTGCATCATTTG | 58.2 |
| *RAC1* Reverse primer | GATGGGAGTGTTGGGACAGT | 59.8 |
| *CDC42* Forward primer | AGCGATTCTCCTGCCTCAG | 60.7 |
| *CDC42* Reverse primer | CGCCCACAACAACACACTTA | 60.6 |
| *NFE2L1* Forward primer | GTCCCTGGAAGAGTGCCTTAG | 60.1 |
| *NFE2L1* Reverse primer | CCGGTCAGAAGAGGAGACAA | 60.5 |
| *NFRKB* Forward primer | CAGCAGCGCTACTTGAAGGT | 60.7 |
| *NFRKB* Reverse primer | ATCCGTGGTTGAAAGTGTGG | 60.8 |
| *HOXA2* Forward primer | CCTGGATGAAGGAGAAGAAGG | 60.2 |
| *HOXA2* Reverse primer | GCTGTGTGTTGGTGTAAGCAGT | 60.3 |
| *PSMB5* Forward primer | CAGAAGAGCCAGGAATCGAA | 60.5 |
| *PSMB5* Reverse primer | GCCTAGCAGGTATGGGTTGA | 60.1 |
| *GTF2A2* Forward primer | ACTGGCTCAGAGGGTCAGG | 60.4 |
| *GTF2A2* Reverse primer | AGTCCACACATTATCGCAGAATC | 60.4 |

# Supplementary Figures


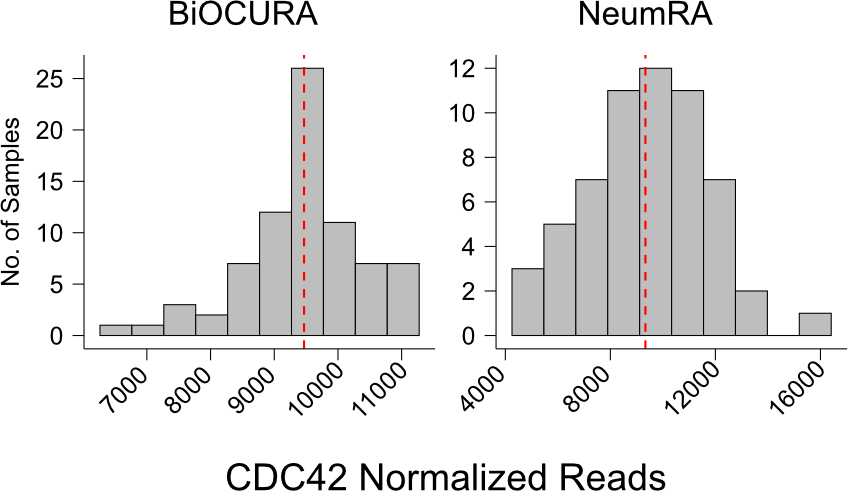


**Supplementary Figure S1. Distribution of *CDC42* expression levels in CD14^+^ cells in the BiOCURA and NeumRA cohorts.** Histogram of CD14^+^ cells by expression of *CDC42* normalized reads (X-axis), absolute number of samples (Y-axis). Red line indicates division into *CDC42^hi^* and *CDC42^lo^* groups.

**
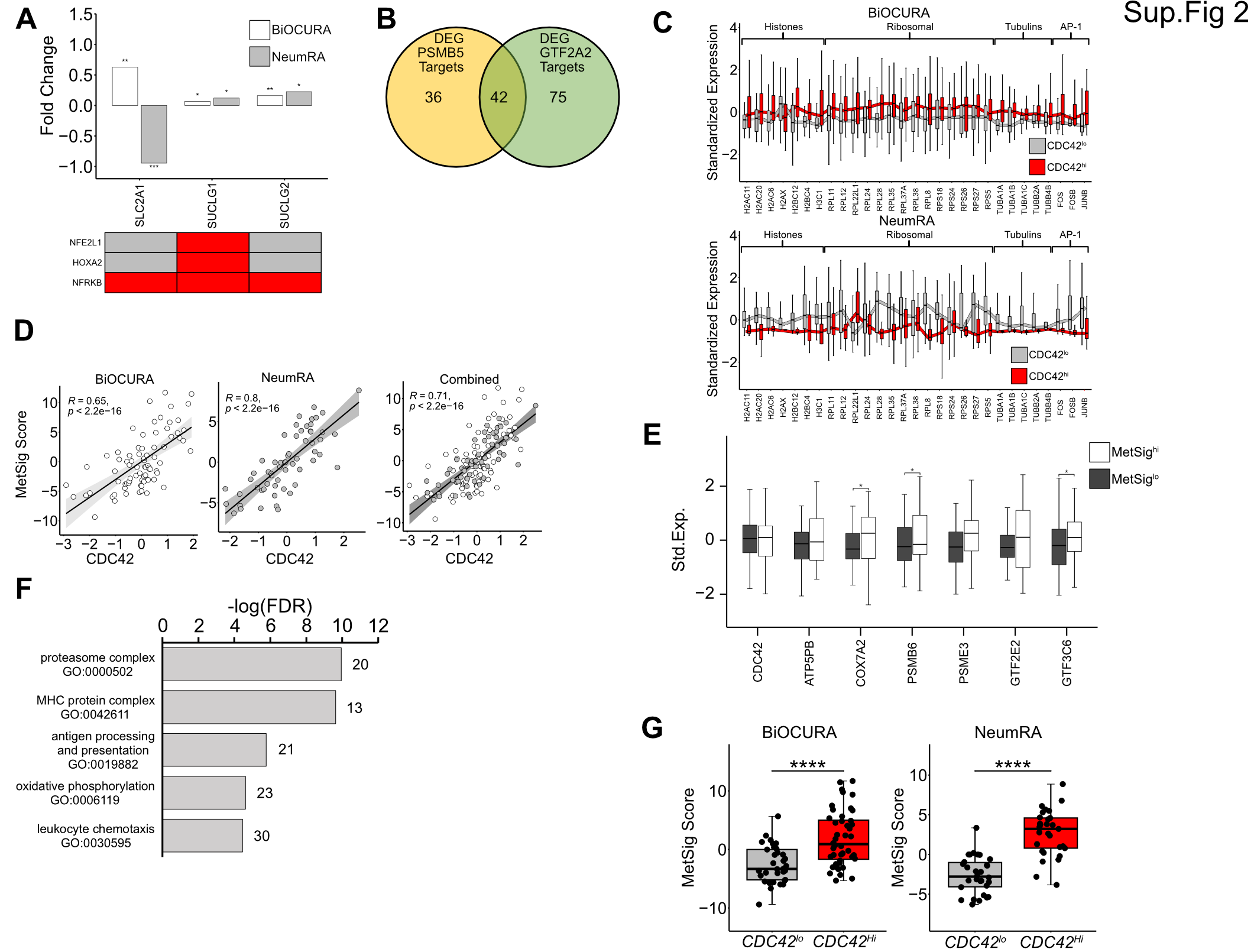
 Supplementary Figure S2. *CDC42*-related metabolic signature of CD14^+^ cells.** **(A)** Bar plot of transcriptional difference (fold change, FC) in GLUT1 gene *SLC2A1* and succinyl-CoA ligase genes *SUCLG1* and *SUCLG2* between *CDC42*^hi^ and *CDC42*^lo^CD14^+^cells in BiOCURA (white) and NeumRA (grey) cohorts. Heatmap below indicates transcriptional regulation by, NFE2L1, HOXA2 or NFRKB. P-values are calculated by DESeq2 test (*) indicates p-value<0.05. (**) indicates p-value<0.01. **(B)** Venn diagram of DEG targets for GTF2A2 and PSMB5. **(C)** Box plot of expression of genes transcriptionally controlled by GTF2A2 and PSMB5 in *CDC42*^hi^ (red) and *CDC42*^lo^ (white) CD14^+^ cells in BiOCURA (n=77) and NeumRA (n=77) cohort. **(D)** Scatter plot of Spearman correlation between the metabolic signature (MetSig) and *CDC42* expression of BiOCURA (white dots), NeumRA (grey dots) cohorts individually and combined (n=136). Spearman regression line with 95 % confidence indicated by area. **(E)** Box plot of gene expression for *CDC42* and MetSig genes in MetSig^hi^ (white) and MetSig^lo^ (grey) CD14^+^ cells. P-values are calculated by DESeq2 test (*) indicates p-value<0.05. **(F)** Bar plots of the false discovery rate (FDR) for the GO biological processes enriched in MetSig^hi^CD14^+^ cells. Intersect size by gene number is displayed. **(G)** Box plots of MetSig score between *CDC42^lo^*CD14^+^ and *CDC42^hi^*CD14^+^ in BiOCURA and NeumRA cohort. P-values are calculated by Mann-Whitney test, (****) indicates p-value<0.0001.


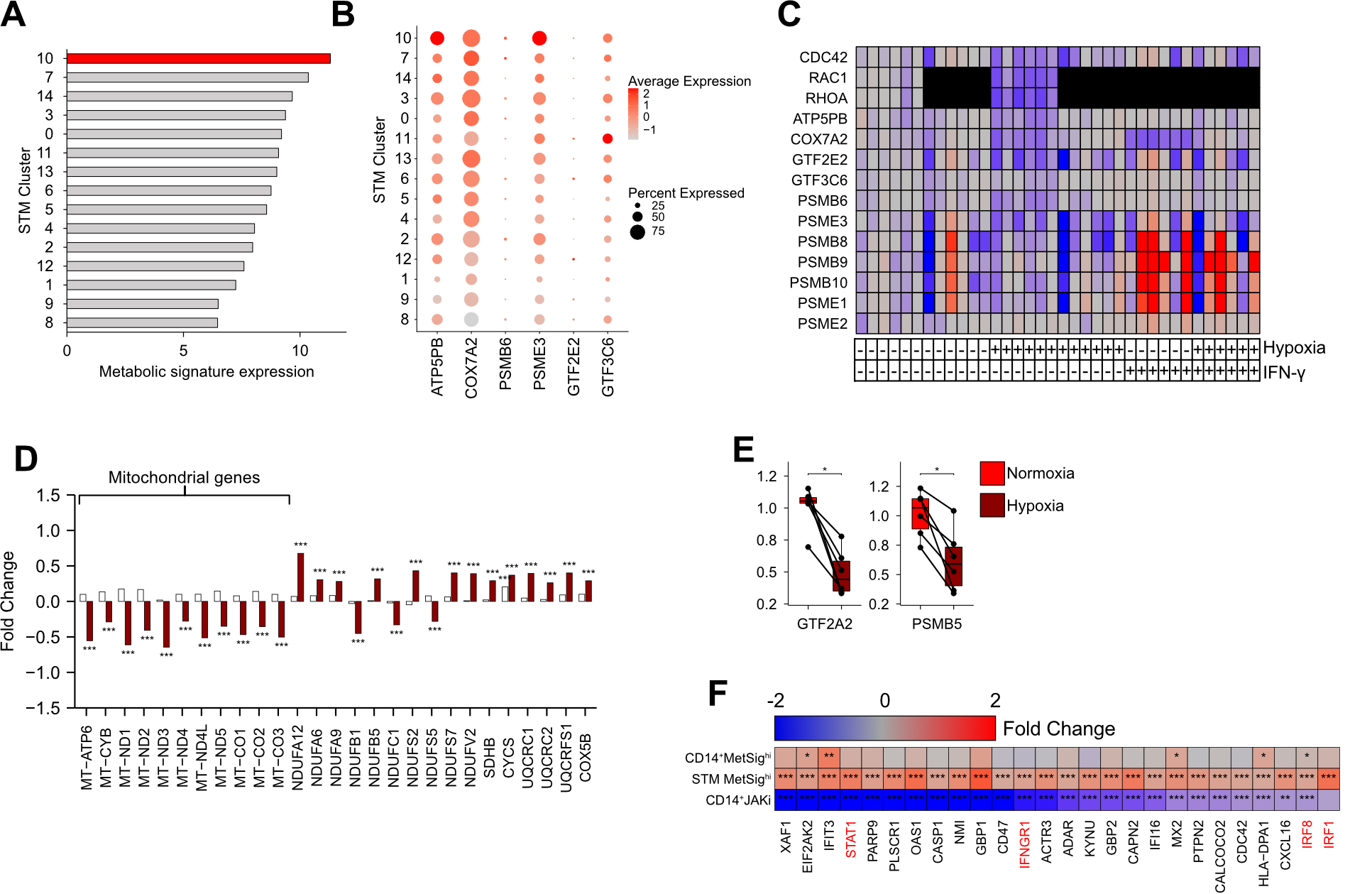


**Supplementary Figure S3**. ***CDC42^hi^MetSig^hi^* cluster of synovial tissue macrophages (STM). (A)** Bar blot of the MetSig in STM clusters derived from scRNAseq. **(B)** Dot plot of individual expression of the MetSig genes in clusters. **(C)** Heatmap of the gene expression difference in RQ between CD14^+^ cells (n=12) cultured in hypoxic conditions with IFN-γ (0 and 50 ng/ml). Expression is normalized to CD14^+^ cells cultured in normoxic conditions without IFN-γ. P-values are calculated by the paired Mann-Whitney test**. (D)** Bar plot of the gene expression difference in fold change (FC) between MetSig^hi^ and MetSig^lo^ CD14^+^ cells (open bars) and between MetSig^hi^STM and other STM clusters (red bars). **(E)** Box plot of *GTF2A2* and *PSMB5* mRNA in CD14^+^ cells (n=6) cultured in normoxic and hypoxic (1% O_2_) conditions. Relative quantity (RQ) in relation to *ACTB* gene. P-values are calculated by Wilcoxon signed rank test (*) indicates p-value<0.05. **(F)** Heatmap of expression difference in IFN-sensitive genes between MetSig^hi^ and MetSig^lo^CD14^+^cells (upper row), MetSig^hi^STM cluster compared to remaining clusters (middle row), and CD14^+^ cells of JAKi-treated patients compare to the other treatments (bottom row).


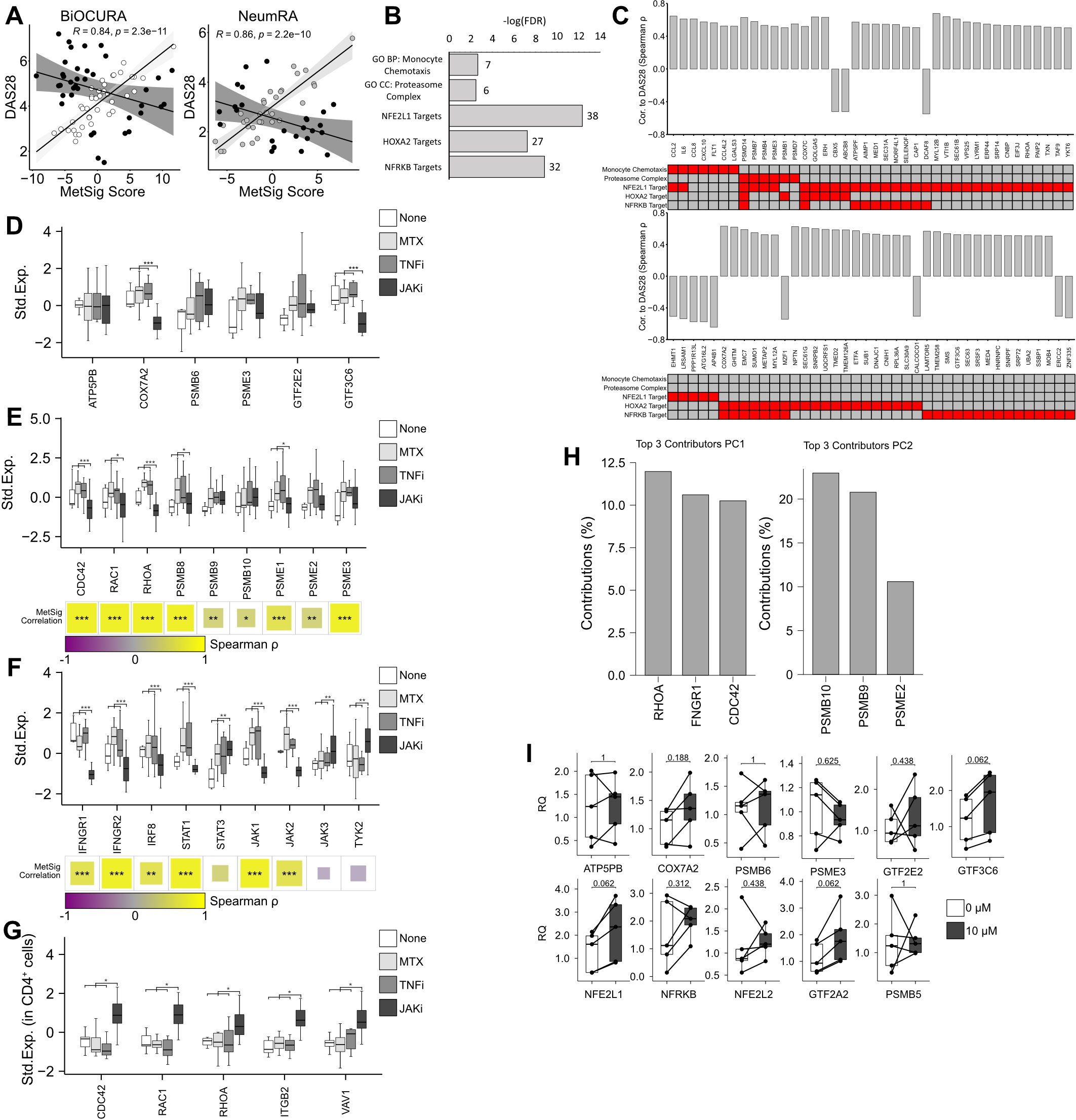


**Supplementary Figure S4**. **Treatment with JAK-inhibitors suppress immunoproteasome and antigen presentation in CD14^+^ cells. (A)** Scatter plot of the Spearman correlation between RA disease activity (DAS28) and metabolic signature (MetSig) in BiOCURA and NeumRA cohort. Spearman regression line with 95 % confidence indicated by area. Black dots indicate the samples outside the model. **(B)** Bar plots of the false discovery rate (FDR) for the GO biological processes and transcriptional regulators enriched in genes correlating to DAS28 in the samples within the model (n=67). **(C)** Bar plot of Spearman ρ correlation between gene expression and DAS28 for the enriched genes. Heatmap below indicates transcriptional regulation by, NFE2L1, HOXA2 or NFRKB as well as involvement in GO: *Monocyte Chemotaxis* or GO: *Proteasome Complex* **(D-G)** Box plot of the gene expression in CD14^+^ cells of patients with different treatment. MTX, methotrexate (n=18); TNFi, TNF-α inhibitors (n=10); JAKi, JAK inhibitors (n=24), none (n=7) **(D)** MetSig genes, **(E)** Rho-GTPases and immunoproteasome. **(F)** IFN/JAK/STAT-pathway genes. **(G)** Rho-GTPases, *ITGB1* and *VAV2* in CD4^+^ cells. Heatmap of Spearman correlation to MetSig is shown below. P-values are calculated with DESeq2 between JAKi treated (n=24) and TNFi, MTX, non-treated patients (n=35). (*) indicates p-value<0.05 (**) p-value<0.01 and (***) p-value<0.001. **(H)** Barplot of top 3 contributors of PC1 and PC2 and their percentage contribution. **(I)** Box plot of transcription change in CD14^+^ cells treated with tofacitinib (0 and 10 µM). P-values are calculated by Wilcoxon signed rank test.
